# DOES SOCIAL ACTIVITY PARTICIPATION MODERATE THE RELATIONSHIP BETWEEN AGE AND COGNITION? EVIDENCE FROM THE HRS

**DOI:** 10.1093/geroni/igae098.3044

**Published:** 2024-12-31

**Authors:** Layla Katharine Santana, Hongdao Meng, Lindsay Peterson, Minh Quan Le, Mingyang Li, Debra Dobbs

**Affiliations:** University of South Florida, Tampa, Florida, United States; University of South Florida, Tampa, Florida, United States; University of South Florida, Tampa, Florida, United States; University of South Florida, Tampa, Florida, United States; University of South Florida, Tampa, Florida, United States; University of South Florida, Tampa, Florida, United States

## Abstract

Active participation in social activities contributes to increased social engagement and may have a positive impact on cognitive health in later life. Despite the well-documented association between social activity and cognitive function, the potential moderating role of social activity in age-related cognitive decline has not been well understood. In this cross-sectional study, we used data from the Health and Retirement Study (HRS), a nationally representative dataset of U.S. adults aged 50 and above (n=9,919), to examine the hypothesis that social activity participation moderates the relationship between age and cognition. Cognition was assessed by using a 27-point cognitive functioning. Social activity participation was measured by a validated 10-point scale, which measures self-reported activity participation. We estimated multivariable linear regression models in which cognitive functioning was regressed on age, and social activity participation while controlling for individual characteristics and potential confounders. We used the Hayes conditional process analysis and Johnson-Neyman plot to test the hypothesized relationships. The average age of the study sample was 67 years (SD=10.27), 59.5% were female, and 66.1% were non-Hispanic White. As expected, age was negatively associated with cognition (β = -0.924; p <.001), and social activity participation was positively associated with cognition (β = 0.122; p <.001). The interaction between age and social activity participation was statistically significant (β = 0.0409; p = 0.005), indicating a significant moderating relationship. These findings suggest that social engagement may be a protective factor. Future studies should examine whether the findings can be replicated in a longitudinal study.

